# NMR, Novel Pharmacological and In Silico Docking Studies of Oxyacanthine and Tetrandrine: Bisbenzylisoquinoline Alkaloids Isolated from *Berberis glaucocarpa* Roots

**DOI:** 10.1155/2018/7692913

**Published:** 2018-05-16

**Authors:** Muhammad Alamzeb, Muhammad Omer, Mamoon Ur-Rashid, Muslim Raza, Saqib Ali, Behramand Khan, Asad Ullah

**Affiliations:** ^1^Faculty of Sciences, Department of Chemistry, University of Kotli, Kotli 11100, Azad Jammu and Kashmir, Pakistan; ^2^Institute of Chemical Sciences, University of Peshawar, Peshawar 25120, Pakistan; ^3^Institute of Chemical Sciences, University of Swat, Swat 19201, Pakistan; ^4^Department of Chemistry, Baluchistan University of Information Technology, Engineering and Management Sciences (BUITEMS), Takatu Campus, Quetta 87100, Pakistan; ^5^State Key Laboratory of Chemical Resource Engineering, College of Life Science and Technology, Beijing University of Chemical Technology, Beijing, China; ^6^Department of Chemistry, Islamia College University, Peshawar 25000, Khyber Pakhtunkhwa, Pakistan

## Abstract

Urease enzyme is responsible for gastric cancer, peptic ulcer, hepatic coma, and urinary stones in millions of people across the world. So, there is a strong need to develop new and safe antiurease drugs, particularly from natural sources. In search for new and effective drugs from natural sources bioassay-guided fractionation and isolation of *Berberis glaucocarpa* Stapf roots bark resulted in the isolation and characterization, on the basis of 1D and 2D NMR data, of two bisbenzylisoquinoline alkaloids, oxyacanthine (**1**) and tetrandrine (**2**), followed by urease inhibition studies. Crude extract, all the subfractions and the isolated compounds **1** and **2** displayed excellent urease enzyme inhibition properties *in vitro*. The antiurease nature and possible mode of action for compounds **1** and **2** were verified and explained through their molecular docking studies against jack-bean urease enzyme. Half-maximum inhibitory concentration (IC_50_) was calculated for compounds **1** and **2**. The IC_50_ value was found to be 6.35 and 5.51 *µ*g/mL for compounds **1** and **2**, respectively. Both compounds **1** and **2** have minimal cytotoxicity against THP-1 monocytic cells.

## 1. Introduction

The genus *Berberis* (Berberidaceae) is represented by about 500 species distributed almost all over the world but mainly in Pakistan, India, Japan, China, Central and West Asia, Southeast Asia, Europe, East Africa, and North America [1]. The genus *Berberis* is known to possess isoquinoline, bisbenzylisoquinoline, protoberberine, aphorphine, benzyl isoquinoline, and protopine alkaloids [2]. The phytochemical screening of the crude extracts of *Berberis* species have revealed the presence of alkaloids, flavonoids, steroids, glycosides, terpenoids, reducing sugars, and saponins [3]. Recent pharmacological studies have established diverse pharmacological properties for different members of the genus *Berberis* including antimicrobial, antinociceptive, anti-inflammatory, antihistaminic, anticholinergic, vasodilatory, antipyretic, antibacterial, antifungal, antirheumatic, and alleviating urinary and gastrointestinal discomforts. The presence of protoberberine and bisbenzylisoquinoline alkaloids like berberine, oxyberberine, palmatine, oxyacanthine, berbamine, and tetrandrine are mainly responsible for the diverse pharmacological properties of the genus [4, 5].

Urease (urea amidohydrolases) is a major contributor to the pathologies induced by *H. pylori*. The active sites of urease enzyme are nickel ions (Ni^2+^) and sulfhydryl groups, and they are responsible for the catalytic effects of the urease enzyme. Urease is responsible for the hydrolysis of urea which produces ammonia which then neutralizes the acid produced in stomach, thus producing a pH environment conducive for the survival and colonization of *H. pylori* bacterium [6]. Urease causes a variety of human diseases such as peptic and duodenal ulcers and gastric carcinoma. Berberine and palmatine, isoquinoline alkaloids from *Berberis*, have already shown antiurease properties [7, 8]. Bisbenzyl isoquinoline alkaloids have been reported to possess biological properties like antimicrobial, cardiovascular, immunomodulatory, antiblood coagulant, cytotoxic effects, and antituberculosis [9, 10]. Tetrandrine and oxyacanthine have been found to have antioxidant, antidiabetic, antimicrobial, anticancer, antihair loss, anti-inflammatory, antiallergic, anticalcium channel blocker, antihypertensive, sleep enhancing, and anticancer effects [11–13].

Thus keeping in mind the broad spectrum of pharmacological properties of *Berberis* and its bisbenzyl isoquinoline alkaloids, we explored *Berberis glaucocarpa* crude extract, subfractions, and oxyacanthine (1) and tetrandrine (2) for their *in-vitro* antiurease enzyme properties against jack-bean urease enzyme and molecular docking studies.

## 2. Materials and Methods

### 2.1. General Procedures

Merck Kieselgel silica gel 60 PF_254_ (70–230 mesh ASTM) and Sephadex LH-20 were used for column chromatography (CC) and thin layer chromatography (TLC). Melting points were determined on Stuart digital melting point apparatus (SMP 10) and are uncorrected. Ultraviolet spectra were recorded on Thermo Spectronic Unicam UV-300. Infrared spectra were recorded on JASCO FTIR-4200A and Thermo Scientific FTIR Nicolet-380. Electron impact ionization mass spectrometry (EIMS) was carried out with JEOL MS-Route instrument. High-resolution electrospray ionization mass spectrometry (HR-ESIMS) was performed on Thermo Scientific Exactive LCQ Fleet instrument. ^1^H NMR, ^13^C NMR, and 2D NMR were recorded on Bruker Avance DRX-400 and 500 MHz and JEOL 400 MHz instruments.

### 2.2. Plant Material

*Berberis glaucocarpa* Stapf was collected from Azad Kashmir Pakistan during flowering period and was identified by Professor Dr. Tanveer Akhtar (Chairperson Botany Department University of Azad Jammu and Kashmir). Voucher specimen number 9615-A was deposited in the herbarium of Botany Department University of Peshawar.

### 2.3. Extraction and Isolation

Root bark (4 kg) was first dried at room temperature, pulverized, and then extracted with commercial grade methanol. This upon concentration with rotary evaporator yielded a dark brownish-black residue (296 g). The residue was then treated with 5% aqueous HCl solution to afford Fraction A (107 g). The filtrate was then extracted with dichloromethane to afford Fraction B (16 g). The acidic filtrate was then basified with NH_3_ to pH 9 and extracted with ethyl acetate to afford Fraction C (59 g). The remainder aqueous solution was termed as Fraction D, which was a mixture of quaternary alkaloids. Fraction **A** was found to mostly consist of isoquinoline alkaloid berberine and was therefore not pursued further. Fraction **B** also consists of nonalkaloids and protoberberine alkaloids and hence was not pursued further.

Fraction **C** (20 g) was subjected to silica gel column chromatography using a mixture of n-hexane-ethyl acetate (100 : 0 to 40 : 60), dichloromethane-methanol (100 : 0 till 60 : 40), and then chloroform-methanol (100 : 0 to 80 : 20) to afford a total of 278 subfractions (**1** **FC to 278** **FC**) which were combined to 11 major subfractions (**1** **CFC to 11** **CFC**) after performing comparative TLCs for all of them.

Subfraction **3** **CFC** (891 mg) was subjected to column chromatography over Sephadex LH-20, and the column was eluted with a mixture of methanol and chloroform (50 : 50). After repeating CC over Sephadex LH-20 using the same solvent system (chloroform: methanol; 50 : 50), finally compound **1** (33 mg) was obtained as off-white amorphous powder.

Subfraction **8** **CFC** (674 mg) was subjected to column chromatography (CC) over silica gel, and the column was eluted with a mixture of chloroform and methanol (90 : 10) to afford 46 minor fraction (8CFC1–8CFC46) which were combined on the basis of comparative thin layer chromatography to 5 major fractions **(8CFCM1–8CFCM5)**.

Major fraction **8CFCM2** (96 mg) was subjected to CC over Sephadex LH-20, and it was eluted with a mixture methanol and dichloromethane (60 : 30) to yield colorless semicrystalline solid compound **2** ( 21 mg).

#### 2.3.1. Oxyacanthine

It is off-white amorphous powder (CHCl_3_) with m.p.: 214–216°C and UV (MeOH) *λ*_max_ (log *ε*): 281 (5.38) nm. IR ῡ: 3313 (O-H, st.), 2921 (C-H, st. sat.), 2831 (C-H, st. *N*-methyl), 1607, 1516 (C=C st. aromatic.) cm^−1^, ^1^H NMR (400 MHz, CDCl_3_), and ^13^C NMR (100 MHz, CDCl_3_) (Table 1). HR-ESIMS = 609.2958 [M + H]^+^ (calculated for C_37_H_40_N_2_O_6_ = 608.2886). EI-MS: *m*/*z* 608 [M^+^] (calculated for C_37_H_40_N_2_O_6_). EI-MS: *m*/*z* (rel. int. %): 608 (M^+^, 100), 593 (29), 577 (6), 416 (12), 395 (81), 381 (69), 379 (28), 365 (10), 335 (6), 198 (51), 192 (17), 174 (31), and 146 (8).

#### 2.3.2. Tetrandrine

It is a colorless semicrystalline solid; UV (MeOH) *λ*_max_ (log *ε*): 282.5 (5.43) nm. IR ῡ: 3370 (O-H, st.), 2953 (C-H, st. sat.), 2840 (C-H, st. *N*-methyl), and 1501 (C=C st. aromatic.) cm^−1^; ^1^H NMR (400 MHz, CDCl_3_) and ^13^C NMR (100 MHz, CDCl_3_) (Table 2).

EI-MS: *m*/*z* 622 [M^+^] (calculated for C_38_H_42_N_2_O_6_). EI-MS: *m*/*z* (rel. int. %): 622 (M^+^, 12), 608 (100), 593 (29), 577 (5.8), 551 (2), 483 (2), 416 (12), 395 (81), 381 (70), 379 (29), 365 (10), 335 (7), 198 (51), 192 (17), 174 (32), 146 (8), 107 (4), 83 (4), 57 (4), and 44 (13).

### 2.4. Culture of THP-1 Cells

**Figure 1:**
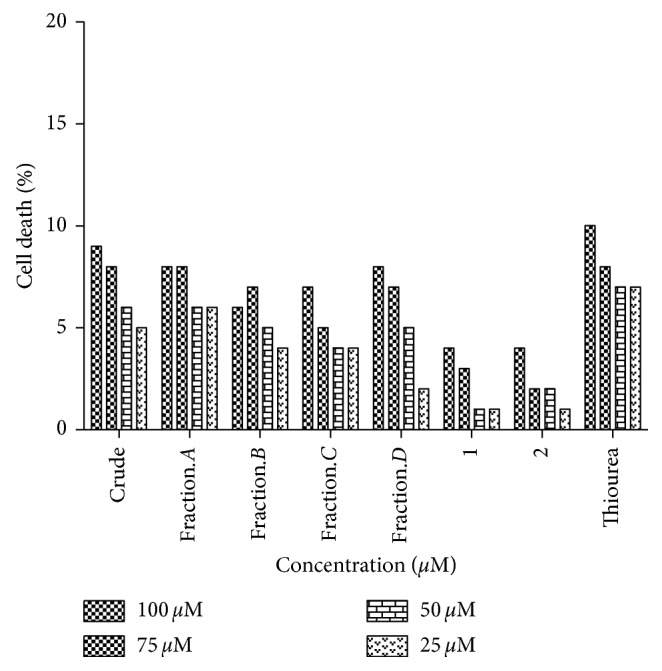
Cytotoxicity results of *Berberis glaucocarpa* and compounds 1 and 2.

THP-1 cells were initially cultured in complete RPMI1640 medium. The cells were grown in carbon dioxide incubator in humidified air with 5% CO_2_ at 37°C. The cells were added with fresh medium on every third day, and after bulking up, they were harvested for checking the cytotoxicity of compounds **1** and **2** (Figure 1).

### 2.5. THP-1 Cytotoxicity of *Berberis glaucocarpa* Stapf

For the determination of cytotoxicity of the tested alkaloids, THP-1 monocytic cells were used. THP-1 cells were originally obtained from London School of Hygiene and Tropical Medicine. THP-1 cells were maintained in RPMI1640 medium (containing 10% HI-FCS, 2 mM L-glutamine, 1% penicillin, and streptomycin) with 5% CO_2_ at 37°C temperature. THP-1 cells with 100 percent viability were centrifuged at 1200 rpm, and the supernatant was removed. Fresh medium was added to the cells, and 50,000 cells/200 *µ*L medium/well were added to flat bottom 96-well plate. For optimization, each compound and the standard (thiourea) were applied in triplicates with four different concentrations (100, 75, 50, and 25 *µ*M). The plate was then placed in a humidified CO_2_ incubator at 37°C. Viability of cells was determined by using the trypan blue exclusion technique after 48 hours via improved neubauer haemocytometer. Viability of cells was determined by trypan blue exclusion analysis before and after treatment with the drugs. An equal volume of 0.4% trypan blue reagent was added to the cells suspension, and the percentage of viable cells was evaluated under dark field microscope. The assay is based on the principle that viable cells are nonpermeable for the dye while the dead cells lost their membrane property and turned blue. Viability was calculated using the following formula:

(1) $$\begin{array}{c} \text{Viability}\left(\%\right)=\left(\frac{\mathrm{live}\;\;\mathrm{cell}\;\;\mathrm{count}}{\mathrm{total}\;\;\mathrm{cell}\;\;\mathrm{count}}\right)\times 100. \end{array}$$

### 2.6. Urease Inhibitory Activity

Twenty-five microlitres (jack-bean urease) of enzyme solution and 55 *μ*L buffers containing 100 mM urea were incubated with 5 *μ*L of test samples (0.5 mM concentration) at 30°C for 15 min in 96-well plates. Indophenol method was used to determine the urease activity by measuring the production of ammonia [14]. Briefly, 45 *μ*L of each phenol reagent (1% *w*/*v* phenol and 0.005% *w*/*v* sodium nitroprusside) and 70 *μ*L of alkali reagent (0.5% *w*/*v* NaOH and 0.1% active chloride NaOCl) were added to each well. The increasing absorbance at 630 nm was measured after 50 min, using a microplate reader (Molecular Devices, USA). All reactions were performed in triplicate in a final volume of 200 *μ*L. The results (change in absorbance per min) were processed by using soft Max Pro software (Molecular Devices, USA). The entire assays were performed at pH 6.8 [15]. Thiourea was used as the standard inhibitor of urease. Percentage inhibitions were calculated by using the following formula:

(2) $$\begin{array}{c} \text{Inhibition}\left(\%\right)=100-\left(\frac{\mathrm{optical}\;\;\mathrm{density}\;\;\mathrm{of}\;\;\mathrm{test}\;\;\mathrm{well}}{\mathrm{optical}\;\;\mathrm{density}\;\;\mathrm{of}\;\;\mathrm{control}\;\;\mathrm{well}}\right)\times 100. \end{array}$$

### 2.7. Docking Simulation

The 3D structure of receptor urease (jack bean) was obtained from protein data bank (PDB) with a four-letter code of 4GY7. The receptor structure energy minimization was carried out by the Swiss pdb viewer v4.1.0 program. The pdb files of compounds **1** and **2** and standard thiourea were prepared by using ChemDraw and Avogadro′s software. The docking simulation was carried out by using AutoDock Vina [16] and i-GEMDOCK v 2.1 software [17]. Before starting the compounds docking, the method validation of both software was carried out by redocking of the cocrystallized thiourea in receptor structure. AutoDock Vina was connected with PyRex tools. Water molecules were cleaned from the enzyme structure; hydrogens addition and gasteiger charges calculation were carried out. The receptor, compounds **1** and **2,** and standard thiourea files (pdb) were uploaded in the PyRex tool. The files were converted into pdbqt format. The grid center was identified by cocrystallized ligand of the receptor with a grid box of center *x* = 20, *y* = −55, and *z* = −20 and size of *x* = 25 Å, *y* = 25 Å, and *z* = 25 Å with an exhaustiveness global search algorithm of 8.

i-GEMDOCK v 2.1 software was also used for docking simulation. The docking study was carried out by setting the software at 70 generations per compound and the population size of 200 random individuals. The best docking conformations were carried out twice implemented by the genetic algorithm. The receptor urease binding pocket was recognized with already cocrystallized metal ions at a distance of 12 Å. The scoring function of i-GEMDOCK is composed of fitness = vdW + H-bond + Elec. The terms vdW, H-bond, and Elec. stands for van der Waal energy, hydrogen bonding energy, and electrostatistic energy, respectively.

The predicted docked poses of compounds **1** and **2** against urease enzyme were analyzed by Discovery studio visualizer version 4.0 PyMOL version 1.7.2 and LIGPLOT+ version v.1.4.5 software.

## 3. Results

### 3.1. Structure Elucidation of Alkaloids

**Figure 2:**
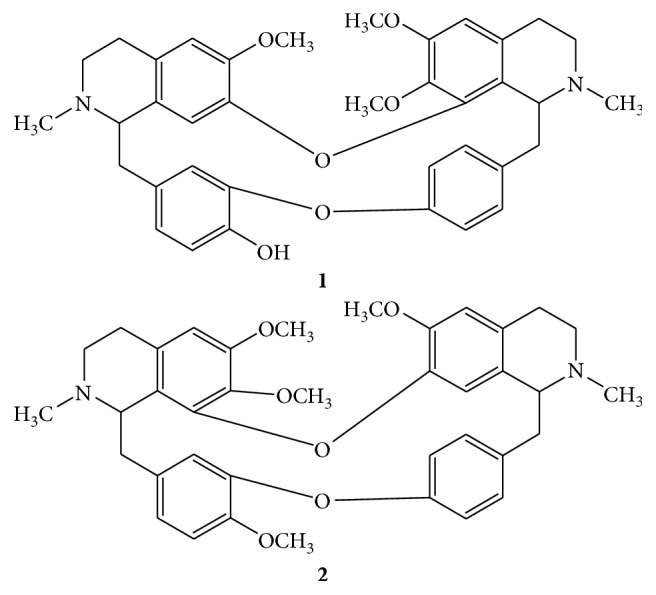
Structures of compounds **1** and **2**.

The structures of bisbenzyl isoquinoline alkaloids (**1**, **2**) were elucidated from their 1D and 2D spectroscopic data and were then confirmed by comparison with literature. The alkaloid **1** was characterized as oxyacanthine [18] while **2** as tetrandrine [19] (Figure 2).

### 3.2. Urease Enzyme Inhibition Effects of Alkaloids **1**-**2**

The urease enzyme inhibition effects of the extract, subfractions, and isolated compounds **1** and **2** are shown in Table 3. Both compounds **1** and **2** showed significant inhibition (IC_50_ = 6.35 and 5.51 *µ*g/mL) as compared to standard thiourea (IC_50_ = 16.39 *µ*g/mL).

### 3.3. Cytotoxicity Studies

Crude extracts, subfractions, and both compounds **1** and **2** displayed minimal cytotoxic results against THP-1 monocytic cells at 25 *µ*M, 50 *µ*M, 75 *µ*M, and 100 *µ*M concentrations (Figure 1).

### 3.4. Docking Studies

Molecular docking studies of urease enzyme were carried out to calculate the inhibiting potency of compounds **1** and **2** by comparison with the standard thiourea. Docking studies were carried out by using two docking software (AutoDock Vina and i-GEMDOCK). Generally, if a compound displays lesser docking score then that compound has good activity.

## 4. Discussion

The current study deals with the chromatographic isolation and spectroscopic characterization of bisbenzylisoquinoline alkaloids **1** and **2** followed by their urease inhibitory effect *in vitro*. Urease (urea amidohydrolase) is typically observed in different bacteria fungi, algae, and plants. Urease catalyzes the hydrolysis of urea to ammonia and carbamate, which is the final step of nitrogen metabolism in living organisms [20]. Carbamate rapidly and spontaneously decomposes to yield a second molecule of ammonia. These reactions may cause significant increase in the pH and are responsible for negative effects of urease activity in human health and agriculture [21, 22]. Infections induced by bacteria such as *Helicobacter pylori* and *Proteus mirabilis* usually have a high urease activity. Urease is vital to *H. pylori* metabolism and virulence. It is necessary for its colonization of the gastric mucosa and is a potent immunogen that elicits a vigorous immune response. This enzyme is used for taxonomic identification and for diagnosis and follow-up after treatment and is a vaccine candidate. Urease represents an interesting model for metalloenzyme studies. *H. pylori* contributes in the urinary tract and gastrointestinal infections and probably augments the severity of several pathological conditions like peptic ulcers and stomach cancer. Ureases are also involved in the development of urolithiasis, pyelonephritis, hepatic encephalopathy, hepatic coma, and urinary catheter encrustation [23, 24]. In this regards, targeting urease for treating pathogenic disorders caused by it may open a new line of treatment for infections caused by urease-producing bacteria. The evaluation of oxyacanthine and tetrandrine for their antiurease was inspired from the use of *Berberis* and its isoquinoline alkaloids, namely, berberine and palmatine against *H. pylori*. The two tested bisbenzyl isoquinoline alkaloids displayed good antiurease properties like berberine and palmatine. Both compounds **1** and **2** displayed better antiurease enzyme inhibition properties than *Berberis glaucocarpa* crude extracts and subfractions which may possibly be due their pure nature as compared to the crude extracts and subfractions. This may be because of the fact that crude extract and subfractions are usually a mixture of different classes of compounds or mixture of different compounds of the same class, and in the mixture form, different compounds may cause to block a certain pharmacological activity of each other. While on the contrary, the antiurease properties of both compounds **1** and **2** were found to be almost similar to each other, which may be due the fact that both of them were pure and are bisbenzyl isoquinoline alkaloids in nature which makes them natural products with broad spectrum of phamacological properties. The minimal cytotoxic values of crude extracts, subfractions, and compounds **1** and **2** against THP-1 monocytic cells are due to the fact that they are natural products (alkaloids) in nature and that is why they are almost perfectly safe for *in-vivo* use.

**Figure 3:**
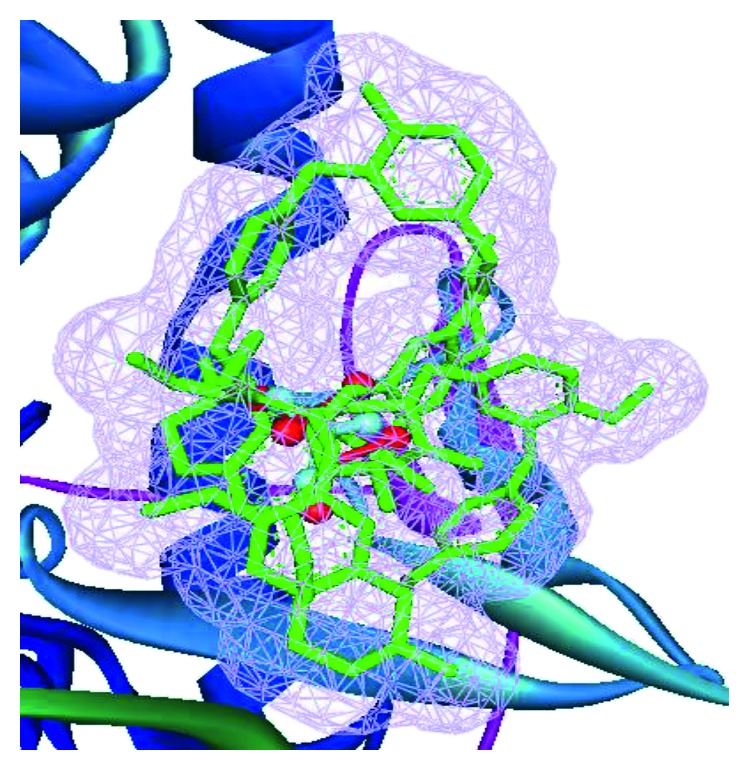
Docked poses of compounds **1** and **2** (sticks with green color) and their superimposition on the cocrystallized thiourea (shown by ball and stick cyan color) and cocrystallized Ni metal ions (indicated by ball and stick red color) against the urease enzyme.

**Figure 4:**
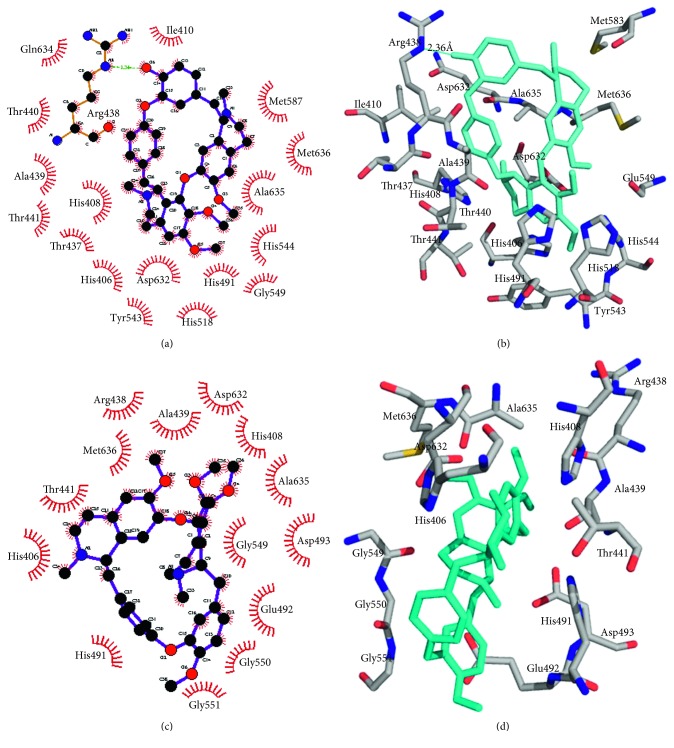
The 2D and 3D schematic representation of binding pocket interactions of the urease enzyme with compounds **1** and **2**.

This analysis of urease enzyme with compounds **1** and **2** is based on the hydrogen bonding and hydrophobic interactions. Biologically, active compounds have the property of making hydrogen bonding and hydrophobic interactions with the enzyme binding pocket. Both compounds **1** and **2** displayed better docking results than the standard thiourea. The predicted docking poses of compounds **1** and **2**, standard thiourea, and cocrystallized Ni atoms of the receptor and their superimposition are presented in Figure 3. The docking score (Table 4) of compound **1** shows that the binding energy is −6.8 kcal/mol (Autodock vina score) while the total energy is −104 kcal/mol (i-GEMDOCK score). These interaction energies are lower than those of the standard thiourea. Compound **1** interaction analysis (Figure 4) revealed that there is only one residue Arg438 involved in the hydrogen bond interaction with a distance of 2.36 Å, while seventeen hydrophobic contacts were observed from the residues His406, His408, Ile410, Thr437, Ala439, Thr440, Thr441, His491, His518, Tyr543, His544, Gly549, Meth587, Asp632, Gln634, Ala435, and Meth636. Compound **2** does not show hydrogen bonding interactions, but the residues of active site of urease enzyme were found to be involved in hydrophobic interactions (Figure 4). These residues include His406, His408, Arg438, Ala439, Thr441, His491, Glu492, Asp493, Gly549, Gly550, Gly551, Asp632, Ala635, and Meth636. In short, it can be concluded that the docking study and *in-vitro* results for compounds **1** and **2** were found to be completely supporting their antiurease nature.

The results of our study showed profound inhibition of urease (jack bean) in concentration-dependent manner. Therefore, our study provided the scientific basis for the traditional uses of *Berberis* as antiulcer.

## Figures and Tables

**Table 1:** ^1^H NMR (400 MHz) and ^13^C NMR (100 MHz) data of compound **1** (CDCl_3_).

Position	*δ*_H_ in ppm (mult., *J* in Hz)	*δ*_C_ in ppm	Position	*δ*_H_ in ppm (mult., *J* in Hz)	*δ*_C_ in ppm
1	3.76, (m)	63.71	1/	3.78, (m)	62.20
N-Me	2.22, (s)	42.70	N/-Me	2.51, (s)	42.75
3	2.85 and 3.39, (m)	45.87	3/	2.54 and 2.97, (m)	44.89
4	2.75 and 2.90, (m)	25.46	4/	2.79 and 2.89, (m)	22.69
4a	—	128.10	4/a	—	127.41
5	6.24, (br. s)	105.46	5/	6.49, (s)	111.23
6	—	151.81	6/	—	149.90
7	—	136.95	7/	—	143.76
6-OMe	3.72, (s)	55.53	6/-OMe	3.55, (s)	55.77
7-OMe	3.08, (s)	60.50			
8	—	147.53	8/	5.95, (br.s)	119.84
8a	—	120.74	8/a	—	128.74
*α*	2.78 and 3.24, (m)	38.56	*α*/	2.79 and 3.26, (m)	37.77
9	—	133.51	9/	—	135.57
10	6.38, (br. s)	114.67	10/	6.40 (dd, 8.6, 2.4)	121.38
11	—	143.53	11/	6.58, (dd, 8.6, 2.4)	121.73
12	—	148.18	12/	—	153.82
13	6.78, (d, 7.4)	115.30	13/	7.08, (dd, 8.6, 2.4)	130.25
14	6.71, (dd, 7.4, 2.2)	123.52	14^/^	7.25, (dd, 8.6, 2.4)	132.25

**Table 2:** ^1^H NMR (400 MHz) and^13^C NMR (100 MHz) data of compound **2** (CDCl_3_).

Position	*δ*_H_ in ppm (mult., *J* in Hz)	*δ*_C_ in ppm	Position	*δ*_H_ in ppm (mult., *J* in Hz)	*δ*_C_ in ppm
1	3.73, (m)	61.51	1/	3.85, (m)	62.84
N-Me	2.28, (s)	43.01	N/-Me	2.59, (s)	42.56
3	2.85 and 3.39, (m)	44.08	3/	2.82 and 3.42, (m)	44.78
4	2.36 and 2.87, (m)	21.92	4/	2.67 and 2.90, (m)	25.69
4a	—	128.02	4/a	—	127.31
5	6.27, (s)	105.46	5/	6.49, (s)	112.10
6	—	151.09	6/	—	149.10
7	—	136.91	7/	—	143.11
6-OMe	3.73, (s)	55.55	6/-OMe	3.37, (s)	55.57
7-OMe	3.15, (s)	60.04			
8	—	147.80	8/	5.98, (br.s)	119.98
8a	—	122.74	8/a	—	128.34
*α*	2.42 and 2.66, (m)	40.86	*α*/	2.68 and 3.25, (m)	38.07
9	—	133.51	9/	—	135.12
10	6.51, (d, 2.2)	116.27	10/	6.28 (dd, 8.2, 2.2)	131.95
11	—	149.51	11/	6.77, (dd, 8.4, 2.4)	121.18
12-OMe	3.85, (s)	55.91	12/	—	153.72
12	—	146.88			
13	6.80, (d, 8.2)	111.31	13/	7.14, (dd, 8.4, 2.3)	121.81
14	6.86, (dd, 8.4, 2.2)	123.07	14^/^	7.35, (dd, 8.4, 2.3)	129.95

**Table 3:** Urease inhibitory activity of *Berberis glaucocarpa* roots and compounds **1** and **2**.

Serial number	Sample	% of inhibition	IC_50_ ± SEM (*µ*g/mL)
1	Crude	82	12.32 ± 0.21
2	Fraction A	91	10.03 ± 0.01
3	Fraction B	54	24.82 ± 0. 43
4	Fraction C	89	11.10 ± 0.71
5	Fraction D	86	11.76 ± 0.19
6	Oxyacanthine (1)	98	6.35 ± 0.34
7	Tetrandrine (2)	99	5.51 ± 0.44
8	Standard (thiourea)	96	16.39 ± 0.11

Values are expressed as mean ± SEM of three different assays.

**Table 4:** The docking results of compounds **1** and **2** and standard (thiourea) against urease enzyme.

Compound	i-GEMDOCK score (kcal/mol)				AutoDock Vina score (kcal/mol)
	Total energy	VDW	H-bond	Elec.	Binding Affinity
1	−104	−93	−11	0	−6.8
2	−97	−85	−12	0	−6.3
Standard thiourea	−44	−24	−20	0	−3.4
